# Blood Pressure Changes After Oral 5-Aminolevulinic Acid Hydrochloride Administered 4–8 h Before TURBT: An Additional Analysis of a Phase III Study (SPP2C102)

**DOI:** 10.3390/life16050819

**Published:** 2026-05-15

**Authors:** Hideo Fukuhara, Makito Miyake, Keita Kobayashi, Atsushi Ikeda, Kent Kanao, Yoshinobu Komai, Ryo Fujiwara, Yusuke Sato, Rikiya Taoka, Keiji Inoue, Kiyohide Fujimoto, Mototsugu Oya

**Affiliations:** 1 Department of Urology, Kochi Medical School Hospital, Nankoku 783-8505, Japan; 2 Department of Urology, Nara Medical University Hospital, Kashihara 634-8522, Japan; makitomiyake@yahoo.co.jp (M.M.); kiyokun@naramed-u.ac.jp (K.F.); 3 Department of Urology, Yamaguchi University Hospital, Yamaguchi 755-8505, Japan; kkoba@yamaguchi-u.ac.jp; 4 Department of Urology, University of Tsukuba Hospital, Tsukuba 305-8576, Japan; scrapsike716@yahoo.co.jp; 5 Department of Uro-Oncology, Saitama Medical University International Medicine Center, Hidaka 350-1298, Japan; kent.kanao2@gmail.com; 6 Department of Urology, Cancer Institute Hospital, Japanese Foundation for Cancer Research, Tokyo 135-8550, Japan; yoshinobu.komai@hadanokitaurology.jp (Y.K.); ryo.fujiwara@jfcr.or.jp (R.F.); 7 Department of Urology, The University of Tokyo Hospital, Tokyo 113-8655, Japan; satoy-uro@h.u-tokyo.ac.jp; 8 Department of Urology, Kagawa University Hospital, Miki 761-0793, Japan; taoka.rikiya@kagawa-u.ac.jp; 9 Center for Photodynamic Medicine, Kochi Medical School, Kochi University, Nankoku 783-8505, Japan; keiji@kochi-u.ac.jp; 10 Department of Urology, Keio University Hospital, Tokyo 160-8582, Japan; moto-oya@z3.keio.jp

**Keywords:** 5-aminolevulinic acid, hypotension, non-muscle invasive bladder cancer, photodynamic diagnosis, transurethral resection of bladder tumor

## Abstract

This study evaluated changes in blood pressure and hypotension-related adverse drug reactions (ADRs) following oral administration of 5-aminolevulinic acid hydrochloride (5-ALA) in patients undergoing transurethral resection of bladder tumor (TURBT). Photodynamic diagnosis under blue light has demonstrated superior sensitivity compared with that of white light when 5-ALA is administered 4 to 8 h before TURBT (95.3% vs. 61.1%, *p* < 0.001); however, data regarding associated blood pressure change remain limited. In this study, blood pressure changes were monitored for 24 h after 5-ALA administration (20 mg/kg) and hypotension-related ADRs were assessed within 14 days post-dose. Both systolic and diastolic blood pressures reached their nadir at 2 h after 5-ALA administration. Absolute blood pressure values were lower in patients with ADRs, whereas the magnitude of blood pressure changes was not different between patients with and without ADRs. The incidence of hypotension, defined as systolic blood pressure ≤ 80 mmHg, was 2.1%, which was lower than that reported in previous retrospective studies (40.0–75.6%). Although hypotension-related ADRs occurred in 25.5% of patients, all events were non-serious and resolved without clinical sequelae. The lower incidence may partly reflect the exclusion criteria and medication restrictions applied in this protocol-controlled trial.

## 1. Introduction

Photodynamic diagnosis (PDD)-assisted transurethral resection of bladder tumor (TURBT) using 5-aminolevulinic acid hydrochloride (5-ALA) is beneficial for treating non-muscle-invasive bladder cancer (NMIBC), as it helps reduce postoperative recurrence risk [1]. The Japanese Urological Association guidelines recommend the use of ALA-PDD (PDD using 5-ALA) as part of the treatment approach [2].

However, following anesthesia administration, hypotension is a notable adverse drug reaction associated with ALA-PDD, necessitating strict perioperative management. Intraoperative systolic blood pressure (SBP) < 80 mmHg or mean artery pressure (MAP) < 60 mmHg are considered thresholds for increased postoperative complication risk [3,4,5,6]. The incidence of hypotension corresponding to SBP < 80 and MAP < 60 during intraoperative bladder cancer TURBT are in the range of 40.0–75.6% [7,8,9,10] and 25.4–70% [11,12], respectively. In neurosurgery, Chung et al. identified a history of hypertension and antihypertensive therapy as significant risk factors for hypotension following 5-ALA administration for brain tumors, with an odds ratio of 17.7 [13]. Multivariate analyses from previous studies identified general anesthesia and a history of hypertension as risk factors for 5-ALA-induced hypotension in patients with bladder cancer [8,14]. Additionally, decision tree analysis classified other potential risks, including age, renal function, and preoperative blood pressure.

Severe 5-ALA-induced hypotension requiring intensive care unit management has occasionally been reported [15,16], necessitating treatment with large doses of vasopressors for extended durations. This underscores the need for heightened awareness and timely intervention by urologists and anesthesiologists.

In Japan, before September 2024, the authorized administration time for oral 5-ALA in PDD of NMIBC was restricted to 2–4 h before TURBT, limiting flexibility in scheduling and procedural convenience. To evaluate whether a wider administration window could maintain sufficient diagnostic performance, the SPP2C102 study assessed the efficacy and safety of PDD when 5-ALA was administered 4–8 h before TURBT. In that study, the sensitivity of PDD was significantly higher than that of white light (95.3% vs. 61.1%, *p* < 0.001) [17], showing a similar trend to that previously reported for administration 2–4 h before TURBT [18], although no direct comparison between the two intervals was performed. Furthermore, no new safety concerns were reported. Based on these efficacy and safety data, the authorized administration window for 5-ALA in Japan was revised to 2–8 h before TURBT through regulatory review, enhancing the practicality of PDD implementation. In this context, the present manuscript focuses on the hemodynamic safety of 5-ALA administration within the currently authorized dosing window.

If the interval between 5-ALA administration and TURBT is altered, the timing of the interaction between blood pressure fluctuations induced by 5-ALA and anesthesia-related blood pressure reduction is expected to change. However, a few studies have reported blood pressure changes and the associated risk of hypotension when 5-ALA is administered more than 4 h before TURBT [19]. Therefore, using data from the SPP2C102 study, the temporal changes in blood pressure before and after 5-ALA administration were analyzed as the primary endpoint. In addition, the incidence and risk factors of hypotension-related adverse drug reactions (ADRs) were also evaluated as the secondary endpoint.

## 2. Materials and Methods

### 2.1. Study Design

This single-arm, multicenter phase III trial was approved by the review board of each institution and conducted from January 2022 to May 2023 in accordance with Good Clinical Practice guidelines and the Declaration of Helsinki. The study protocol was registered under jRCT2061210055.

To mitigate the risk of hypotension, the following exclusion criteria and restrictions on concomitant drugs were established. Patients with uncontrollable hypertension, SBP ≤ 100 or diastolic blood pressure (DBP) ≤ 60 mmHg during screening (from 28 days before administration to the day before administration), and a history of hypotension or decreased blood pressure during TURBT were excluded. Additionally, antihypertensive drugs were prohibited from the day before to the day of 5-aminolevulinic acid hydrochloride (5-ALA) administration (prior to TURBT). To minimize bias in administration timing, patients were randomly assigned in equal proportions to two strata based on different administration periods: stratum 1 (4–6 h before TURBT) and stratum 2 (6–8 h before TURBT). This assignment was automatically performed using an electronic data collection system employing a permutation block method with a predetermined block size (4 patients). The size and sequence of blocks were determined by the block allocators before starting and were not disclosed until the end of study. The investigational agent, 5-ALA, was supplied by SBI Pharmaceuticals (Tokyo, Japan).

### 2.2. Primary Endpoint: Evaluation of Changes in Blood Pressure over Time After Oral Administration of 5-ALA

**Figure 1:**
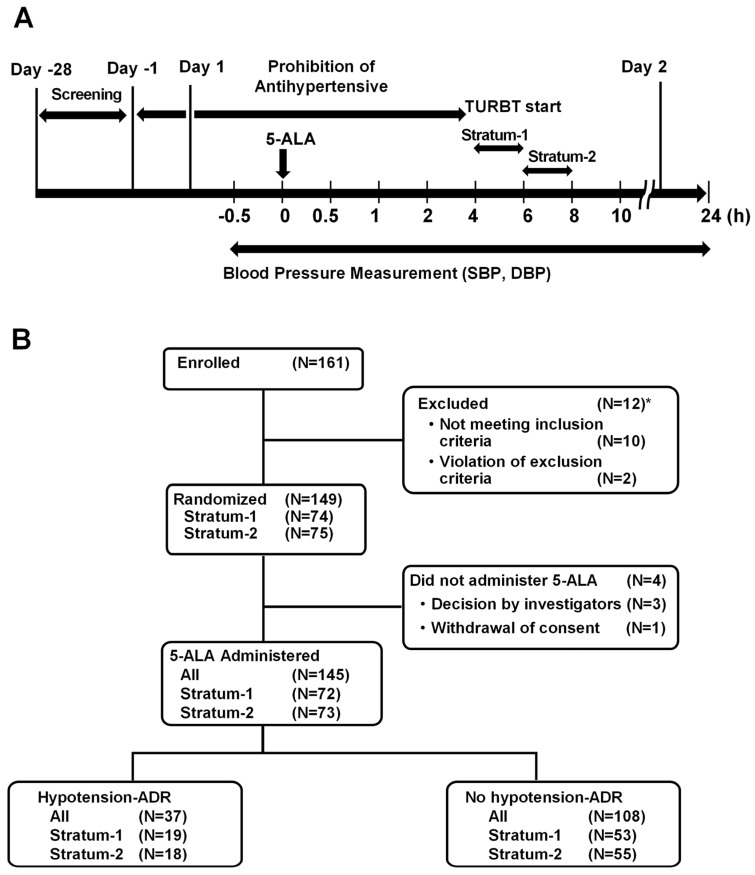
(**A**) Study design. (**B**) Flow of subject groupings. *: One patient was deemed ineligible based on the inclusion and exclusion criteria.

Figure 1 illustrates the study design and patient flowchart. To ensure patient safety, concomitant use of antihypertensive drugs was prohibited from the day before 5-ALA administration until TURBT initiation on the day of administration. DBP and SBP were monitored over time (0.5 h before, and 0.5, 1, 2, 4, 6, 8, 10, 24 h after administration of 5-ALA at 20 mg/kg body weight). Resting blood pressure was measured in a fixed position (e.g., after 5 min in supine position) using a validated sphygmomanometer. In addition, intraoperative vital log data was also used as blood pressure data corresponding to 4 and 6 h for the 1st stratum and at 6 and 8 h for the 2nd stratum.

Changes in blood pressure were evaluated for all patients and for each stratum. Further analysis was performed based on the with or without hypotension-related ADRs.

To evaluate the incidence of hypotension, the proportion of patients with SBP ≤ 80 mmHg was assessed based on criteria established in previous studies [7,8,9,10,19]. It is crucial to maintain SBP > 100 mmHg to reduce the risk of complications [16,17]. Associations between blood pressure fluctuations and various factors, including demographics, procedure, anesthetic, antihypertensive drugs, complications, hematology, blood biochemistry, eGFR, and urinalysis, were analyzed.

### 2.3. Secondary Endpoint: Analysis of Hypotension-Related ADRs Associated with 5-ALA

Any unfavorable events occurring between drug administration and 14 days post-dose, as determined by the physician, were defined as adverse events (AEs) and were encoded using Medical Dictionary for Regulatory Activities version 26.0 and graded according to the Common Terminology Criteria for Adverse Events version 5.0. ADRs were defined as AEs that could not be ruled out as being related to the study drug. Among the ADRs, those associated with hypotension or decreased blood pressure were classified as hypotension-related ADRs.

### 2.4. Statistical Analysis

Statistical analyses specified in the protocol were performed using the Statistical Analysis System (version 9.4), and post hoc analysis in this study was performed using R (version 4.3.1). Fisher’s exact test was applied to compare the categorical variable proportions, whereas a two-sided *t*-test was performed to compare the means of continuous variables. A *p*-value of <0.05 was considered statistically significant. Pearson’s correlation coefficient (R) was used to evaluate associations between SBP and patient background factors. Correlations within the range of 0.2 ≤ |R| < 0.4 were considered weak.

## 3. Results

### 3.1. Primary Endpoint: Evaluation of Changes in Blood Pressure over Time After Oral Administration of 5-ALA

**Figure 2:**
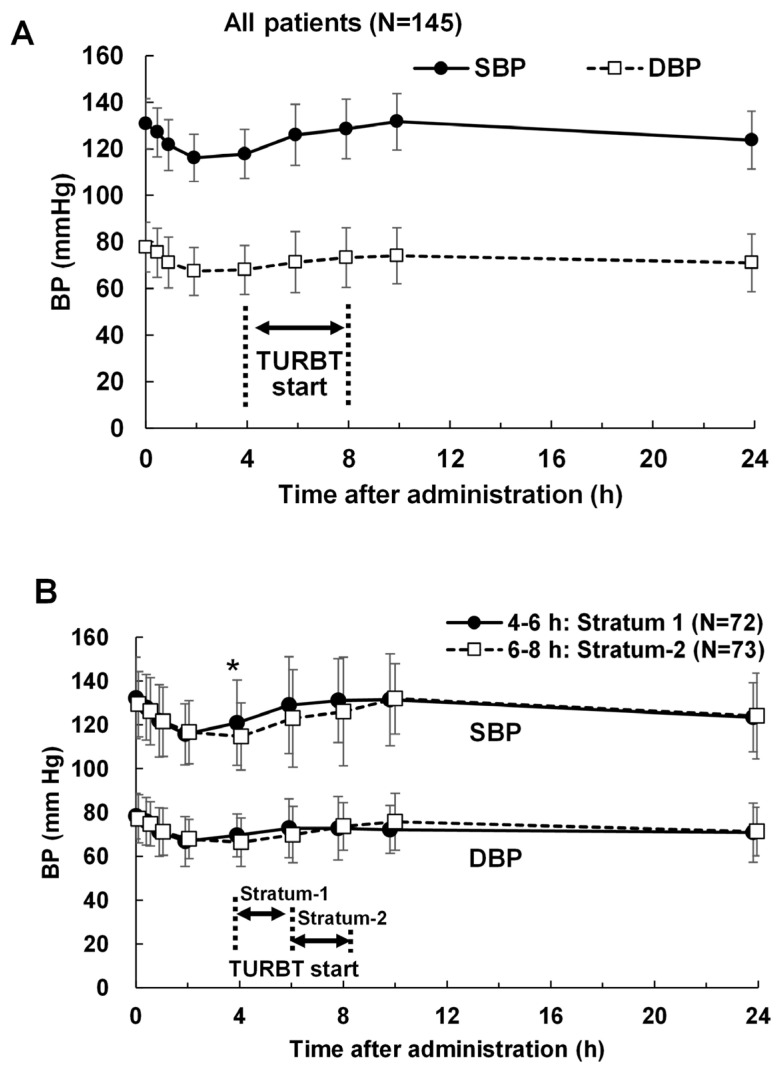
Change in blood pressure after 5-ALA administration. (**A**) Change in blood pressure in all patients. (**B**) Change in blood pressure in each stratum. BP, Blood pressure; SBP, Systolic blood pressure; DBP, Diastolic blood pressure; TURBT, Transurethral resection of bladder tumor. *, *p* < 0.05 (*t*-test).

Figure 2 illustrates blood pressure changes over time after 5-ALA administration. In all patients (n = 145), the mean SBP and DBP reached their lowest values 2 h after administration (Figure 2A). Regarding strata, the largest decrease in mean SBP and DBP occurred at 2 and 4 h after administration for stratum 1 and stratum 2, respectively, with a significant difference in SBP at 4 h (*p* < 0.05, *t*-test, Figure 2B). Thus, both SBP and DBP decreased prior to TURBT.

Table 1 summarizes the timing of the lowest blood pressure recorded. The median time of the lowest blood pressure (SBP/DBP) was 4.00 h/5.53 h for all patients (N = 145), 4.00 h/4.18 h for stratum 1 (N = 72), and 4.00 h/5.67 h for stratum 2 (N = 73). More than 60% of patients recorded their lowest SBP or DBP before TURBT initiation.

**Figure 3:**
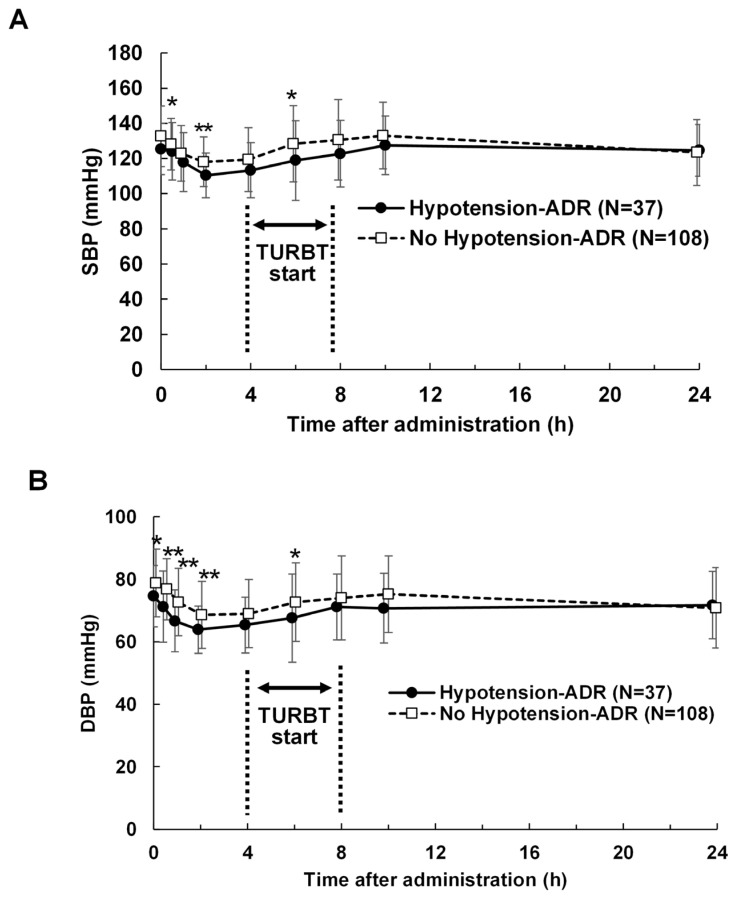
Absolute change in blood pressure after 5-ALA administration with or without hypotension-related adverse drug reactions (ADRs). (**A**) Systolic blood pressure (SBP). (**B**) Diastolic blood pressure (DBP). TURBT, Transurethral resection of bladder tumors; *, *p* < 0.05; **, *p* < 0.01 (*t*-test).

**Figure 4:**
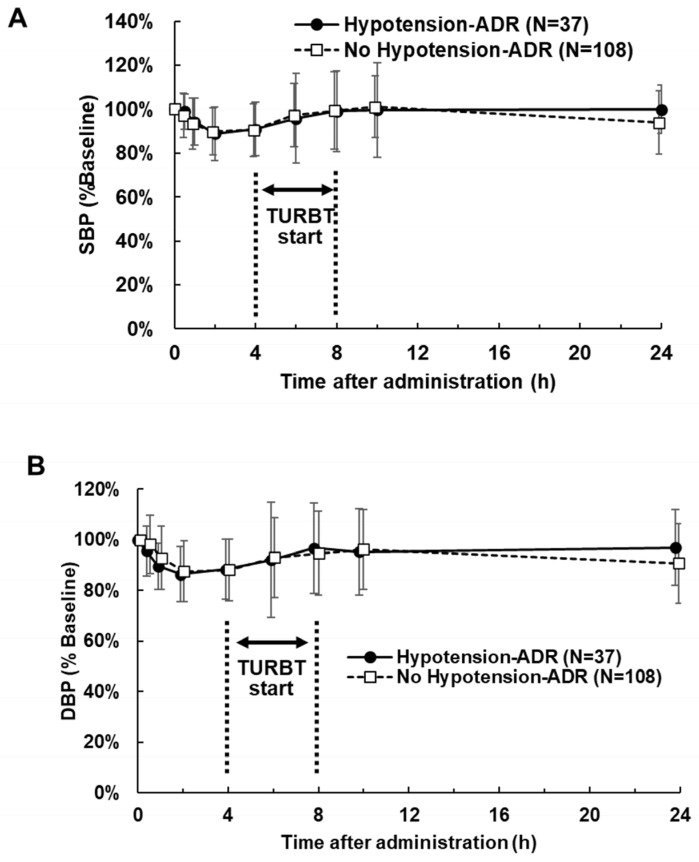
Relative change in blood pressure after 5-ALA administration with or without hypotension-related adverse drug reactions (ADRs). (**A**) Systolic blood pressure (SBP). (**B**) Diastolic blood pressure (DBP). TURBT, Transurethral resection of bladder tumors.

Figure 3 and Figure 4 illustrate the changes in mean SBP and DBP with and without hypotension-related ADRs. In absolute terms (Figure 3), the mean SBP and DBP were lower in patients with ADRs than in those without. However, relative changes in SBP and DBP remained similar regardless of ADR presence (Figure 4).

At the individual level, SBP varied greatly at different time points, with some patients experiencing decreased SBP following TURBT initiation or completion (Figure S1). Among three patients with SBP ≤ 80 mmHg, the time at which SBP reached its minimum coincided with TURBT initiation at 6 h post-dose (cases 2 and 3) or it occurred 1 h after TURBT initiation at 7 h post-administration (case 1).

The correlation between SBP at each time point and patient background characteristics was analyzed to identify factors influencing blood pressure reduction (Figures S2 and S3).

Although weak correlations (0.2 ≤ |R| < 0.4) were observed between SBP and certain patient background factors, no strong correlation (|R| ≥ 0.4) was identified with any of these factors (Figure S2). Among known risk factors for hypotension, hypertension was the only factor that showed a weak positive correlation (R = 0.210–0.365) with higher SBP at multiple measurement points. For other risk factors, weak correlations (0.2 ≤ |R| < 0.4) with SBP were observed only at select measurement points: BMI at 24 h, body weight at 24 h, and sex at 1 h. No clear correlation was observed between SBP and age, type of anesthesia, or use of antihypertensive medications. Regarding relative SBP (% baseline, Figure S3), weak correlations (0.2 ≤ |R| < 0.4) were observed between SBP and several factors. However, none of the known risk factors showed a weak correlation (0.2 ≤ |R| < 0.4) at any measurement point.

### 3.2. Secondary Endpoint: Analysis of Hypotension-Related ADRs Associated with 5-ALA

Figure 1B presents the patient flowchart for this study. Among 161 patients enrolled, 145 patients (stratum 1: N = 72; stratum 2: N = 73) who received 5-ALA were eligible for the safety evaluation after excluding 12 patients who were deemed ineligible and 4 patients who did not receive 5-ALA. Among these, 37 patients (stratum 1: N = 19; stratum 2: N = 18) experienced hypotension-related-ADRs, while 108 patients (stratum 1: N = 53; stratum 2: N = 55) did not.

Table 2 summarizes the incidence of hypotension-related AEs and ADRs reported in this study. The incidence rate of hypotension-related AEs was 39.3% (57/145), of which, 25.5% (37/145) were classified as ADRs. No serious hypotension-related AEs or ADRs were reported. The severity of ADRs was limited to grade 1 (10.3% [15/145]) and grade 2 (15.2% [22/145]), and no grade ≥ 3 ADRs were observed. Regarding outcome, all hypotension-related ADRs resolved, and the majority (34/37) resolved on the day of onset. The remaining three ADRs were resolved either on the day following onset or six days after onset.

The incidence and severity of hypotension-related AEs and ADRs did not differ between the two strata, suggesting that the administration period did not influence hypotension occurrence. Regarding event term, hypotension was the most frequently reported AE (39 AEs in 38 cases [26.2%] and 26 ADRs in 26 cases [17.9%]), followed by a decrease in blood pressure (18 AEs in 18 cases [12.4%] and 10 ADRs in 10 patients [6.9%]) and procedural hypotension (two AEs in two cases [1.4%] and one ADR in one case [0.7%]).

### 3.3. Comparison of Baseline Characteristics Between Patients with and Without Hypotension-Related ADRs

Table 3 and Table 4 compare the baseline characteristics between patients with and without hypotension-related ADRs. The mean ages (±standard deviation [SD]) were 70.3 ± 10.5 and 69.1 ± 9.6 years, respectively; heights were 163.6 ± 9.9 cm and 164.4 ± 8.5 cm; body weights were 63.9 ± 15.5 kg and 64.4 ± 11.6 kg; body mass indices (BMIs) were 23.6 ± 4.2 and 23.7 ± 3.4; SBPs were 131.3 ± 15.5 mmHg and 136.4 ± 14.3 mmHg; and DBPs were 78.9 ± 10.6 mmHg and 81.2 ± 9.7 mmHg for patients with and without ADRs, respectively.

The proportions of men and women with ADRs were 22.9% (27/118) and 37.0% (10/27), respectively. For patients with a history of NMIBC, the proportions were 27.2% (25/92) for primary cases and 22.6% (12/53) for recurrent cases. Based on the Eastern Cooperative Oncology Group Performance Status (PS) scale, the proportions were 26.1% (37/142) for PS 0 and 0.0% (0/3) for PS 1, suggesting a significant decrease in the statistical power.

Regarding hypertension complications, 21.0% (13/62) of patients had hypertension, whereas 28.9% (24/83) did not. Although SBP and DBP were slightly lower in patients with hypotension-related ADRs, no significant differences were observed between groups across all parameters, including the type of anesthesia.

Table 5 presents odds ratios and 95% confidence intervals (CIs) for hypotension-related ADRs based on patient background factors. The odds ratios for the use of antihypertensive medication from the day before administration to the start of TURBT (2.294, 95% CI: 0.489–10.768) and for female sex (1.983; 95% CI: 0.813–4.836) were relatively high but not statistically significant within their respective 95% CIs.

Regarding ECOG PS, the odds ratio could not be calculated because there were no patients with ADRs at PS 1.

## 4. Discussion

Photosensitivity, nausea, vomiting, transient liver dysfunction, ventricular arrhythmias, and hypotension are known ADRs caused by the oral administration of 5-ALA. Severe, prolonged hypotension may necessitate high doses of vasopressors and extended treatment durations, with some cases requiring intensive care unit management [15,16]. In this study, we analyzed hypotension data from the SPP2C102 study. Nohara et al. reported that the frequency of vasopressor use was significantly higher during ALA-PDD-assisted TURBT compared to conventional TURBT [14]. In a multivariate analysis, they found that general anesthesia and regular use of renin-angiotensin system inhibitors were associated with an increased risk of 5-ALA-induced hypotension. Miyakawa et al. investigated the association between severe hypotension following oral administration of 5-ALA and various clinicopathological factors [20]. They identified age ≥ 80 years, BMI ≥ 25, and estimated glomerular filtration rate < 45 as significant risk factors for severe hypotension. The incidence of severe hypotension was 1.1% in patients with 0 or 1 risk factor, 9.7% in those with 2 factors, and 50% in those with all 3 factors. Their findings suggest that severe hypotension results from a combination of these factors rather than a single factor. Specifically, they highlighted that excessive doses of 5-ALA administered to patients with high BMI and decreased metabolic potency due to aging and impaired renal function led to elevated serum 5-ALA concentrations.

PpIX, a metabolite of 5-ALA, is thought to induce vasodilation via increased cGMP levels resulting from sGC activation [21]. Furthermore, heme, metabolized from PpIX in mitochondria, is degraded by heme oxygenase-1, generating carbon monoxide (CO) [22]. Since both cGMP and CO cause vasodilation, the increase in PpIX after 5-ALA administration may contribute to 5-ALA-induced hypotension. Although no reports have described the kinetics of PpIX accumulation in patients with bladder cancer, studies in healthy adults suggest that PpIX accumulation peaks after 4 to 6 h [23,24]. In this study, the peak reduction in blood pressure occurred 2 to 4 h after administration, preceding the peak of PpIX accumulation. This time lag may be attributed to automatic feedback regulation by the sympathetic nervous system [25] or recovery due to vasopressor administration during surgery.

In several studies, SBP ≤ 80 mmHg or <80 mmHg has been used as the threshold to evaluate incidence of 5-ALA-induced hypotension [7,8,9,10,19]. Thus, the incidence of SBP ≤ 80 mmHg in this study was compared with that in previous studies (Table 6). In this study, the incidence of hypotension corresponding to SBP ≤ 80 mmHg was 2.1% (3/145), which was markedly lower than that reported in previous studies, suggesting a lower occurrence of hypotension in this study [7,8,9,10,19].

One possible factor for the low incidence of hypotension in this study is that patients with low baseline blood pressure, as well as those with a history of hypotension or intraoperative blood pressure reduction during previous TURBT procedures, were excluded. However, because of the single-arm design, the relative contributions of patient selection, protocol-defined medication restrictions, and the timing of 5-ALA administration cannot be disentangled, and no causal inference regarding the administration interval can be drawn from the present analysis.

Previous studies, including Yatabe et al. (2020) [11] and the authors’ earlier study [8], have consistently demonstrated that cases in which hypotension occurred tended to have lower preoperative SBP. In this study as well, preoperative blood pressure was lower among patients who experienced hypotension-related ADRs. These findings are consistent with the possibility that excluding patients with low baseline blood pressure may contribute to a lower observed incidence of hypotension. Furthermore, the exclusion of patients with impaired renal or cardiac function, both known risk factors for hypotension, as well as the prohibition of antihypertensive medications on the day of surgery, may have contributed to the reduced frequency of hypotension observed in this study.

Another possible factor for the low frequency of hypotension observed in this study is the timing of anesthesia induction following 5-ALA administration. In this study, the lowest blood pressure was recorded at 2–4 h post-administration of 5-ALA, preceding anesthesia introduction (4–8 h after 5-ALA administration), avoiding the period of lowest blood pressure induced by 5-ALA administration. This timing difference may have mitigated the synergistic hypotensive effects of anesthesia and 5-ALA. Consistent with this hypothesis, Hori et al. reported that the incidence of moderate to severe hypotension was lower when TURBT was performed more than 4 h after administration compared with that when TURBT was performed within 4 h post administration [19]. However, some differences exist in between the results of their retrospective study and this study. For example, regarding the blood pressure trend, in our study, blood pressure decreased 2 h after administration; however, in their study, no decrease in blood pressure was reported until 3 h after administration. Possible factors contributing to this difference include the exclusion criteria for patients at risk of hypotension and the prohibition of concomitant medications in our study.

Although the incidence of hypotension-related ADRs in this study (25.5% [37/145]) was higher than that reported in the SPP2C101 study (1.6% [1/61]) [17], no serious hypotension-related ADRs were observed. Furthermore, most of the hypotension-related ADRs in this study were resolved on the day of onset. In contrast, the SPP2C101 study reported one serious grade 3 ADR [17]. One difference between the protocols of this study and the SPP2C101 trial was that concomitant use of antihypertensive drugs is prohibited from the day before administration. Antihypertensive drugs have been identified as risk factors for hypotension in several retrospective studies. Therefore, the restriction on concomitant use of antihypertensive drugs in this study may be a contributing factor to the absence of serious ADRs.

In addition, several previous studies have reported that general anesthesia is a risk factor for hypotension [8,9,21]. However, in our study, the type of anesthesia did not affect the occurrence of hypotension-related ADRs. The reason for these differences is unknown, but this was probably because patients at high risk of hypotension were excluded from this study.

This study aimed to show non-inferiority of sensitivity under blue light over a prolonged incubation period (4–8 h) compared with that of the previous SPP2C101 study, which had a shorter incubation time (2–4 h). Most protocols in this study were largely consistent with those in the SPP2C101 study, except for the exclusion criteria for hypotension and the prohibition of antihypertensive use, which may reduce the incidence and severity of hypotension.

Several factors may have contributed to the relatively low incidence of severe hypotension observed in this study. These include the exclusion of patients with low baseline blood pressure or prior hypotensive episodes, the temporary prohibition of antihypertensive medications, and the conduct of the trial under closely monitored, protocol-controlled conditions. Another potential factor is that the nadir of blood pressure occurred before anesthesia induction in many patients, thereby avoiding temporal overlap between 5-ALA-related blood pressure reduction and anesthesia-induced hypotension. However, because of the single-arm design, the relative contributions of patient selection, protocol-defined medication restrictions, and the timing of 5-ALA administration cannot be disentangled, and no causal inference regarding the administration interval can be drawn from the present analysis.

Another important limitation is the restricted generalizability of these findings. The SPP2C102 study population represents a carefully selected cohort that may differ substantially from unselected real-world patients, particularly those with baseline hypotension, significant comorbidities, renal dysfunction, or continued antihypertensive therapy. Therefore, the present results should be interpreted as descriptive safety data obtained under defined trial conditions rather than as evidence supporting broader clinical applications. Nonetheless, when clinical trials involving 5-ALA are conducted, the exclusion criteria and restrictions on concomitant medications applied in this study may contribute to risk mitigation and participant safety. Further studies including real-world data or comparative designs are required to better define the independent determinants of hypotension risk and to inform clinical practice.

## Figures and Tables

**Table 1:** Summary of the timing of the lowest recorded blood pressure values.

	All (N = 145)	Stratum-1 (N = 72)	Stratum-2 (N = 73)
SBP minimum time			
Median (range)	4.00 (−0.67~24.33)	4.00 (−0.67~24.33)	4.00 (−0.58~24.17)
Before TURBT initiation, % (patients)	64.1 (93/145)	56.9 (41/72)	71.2 (52/73)
After TURBT initiation, % (patients)	35.9 (52/145)	43.1 (31/72)	28.8 (21/73)
DBP minimum time			
Median (range)	5.53 (−0.48~24.17)	4.18 (−0.43~24.17)	5.67 (−0.48~24.00)
Before TURBT initiation, % (patients)	60.7 (88/145)	51.4 (37/72)	69.9 (51/73)
After TURBT initiation, % (patients)	39.3 (57/145)	48.6 (35/72)	30.1 (22/73)

SBP, systolic blood pressure; DBP, diastolic blood pressure; TURBT, transurethral resection of bladder tumor.

**Table 2:** Summary of hypotension-related AEs and ADRs in SPP2C102 study (N = 145).

	Adverse Events (AEs)		Adverse Drug Reactions (ADRs)	
	Events	Cases (%)	Events	Cases (%)
All (N = 145)	59	57 (39.3%)	37	37 (25.5%)
Serious Events	0	0 (0.0%)	0	0 (0.0%)
Grade 1	21	20 (13.8%)	15	15 (10.3%)
Grade 2	36	36 (24.8%)	22	22 (15.2%)
Grade 3	2	2 (1.4%)	0	0 (0.0%)
Stratum 1 (N = 72)	32	31 (43.1%)	19	19 (26.4%)
Grade 1	8	8 (11.1%)	6	6 (8.3%)
Grade 2	24	24 (33.3%)	13	13 (18.1%)
Grade 3	0	0 (0.0%)	0	0 (0.0%)
Stratum 2 (N = 73)	27	26 (35.6%)	18	18 (24.7%)
Grade 1	13	12 (16.4%)	9	9 (12.3%)
Grade 2	12	12 (16.4%)	9	9 (12.3%)
Grade 3	2	2 (2.7%)	0	0 (0.0%)
PT				
Hypotension	39	38 (26.2%)	26	26 (17.9%)
Grade 1	8	8 (5.5%)	6	6 (4.1%)
Grade 2	29	29 (20.0%)	20	20 (13.8%)
Grade 3	2	2 (1.4%)	0	0 (0.0%)
Decreased blood pressure	18	18 (12.4%)	10	10 (6.9%)
Grade 1	12	12 (8.3%)	9	9 (6.2%)
Grade 2	6	6 (4.1%)	1	1 (0.7%)
Grade 3	0	0 (0.0%)	0	0 (0.0%)
Procedural hypotension	2	2 (1.4%)	1	1 (0.7%)
Grade 1	1	1 (0.7%)	0	0 (0.0%)
Grade 2	1	1 (0.7%)	1	1 (0.7%)
Grade 3	0	0 (0.0%)	0	0 (0.0%)

MedDRA/Ver. 26.0, grading according to CTCAE v5.0. MedDRA, Medical Dictionary for Regulatory Activities; AE, adverse event; ADR, adverse drug reaction; PT, preferred term. Note: The discrepancy between AEs (N = 59) and cases (N = 57) resulted from one patient with both hypotension and decreased blood pressure, and another patient with two hypotension events (Grade 1 and Grade 2).

**Table 3:** Comparison of baseline characteristics between patients with or without hypotension-related ADRs.

	Hypotension-Related ADRs (N = 37)	Non-Hypotension-Related ADRs (N = 108)	
	Average ± SD	Average ± SD	*t*-Test
Age (years)	70.3 ± 10.5	69.1 ± 9.6	*p* = 0.5122
Height (cm)	163.6 ± 9.9	164.4 ± 8.5	*p* = 0.633
Weight (kg)	63.9 ± 15.5	64.4 ± 11.6	*p* = 0.8771
BMI	23.6 ± 4.2	23.7 ± 3.4	*p* = 0.9027
SBP (mmHg)	131.3 ± 15.5	136.4 ±14.3	*p* = 0.06818
DBP (mmHg)	78.9 ± 10.6	81.2 ± 9.7	*p* = 0.2343

ADR, adverse drug reaction; SD, standard deviation; BMI, body mass index; SBP, Systolic blood pressure; DBP, Diastolic blood pressure.

**Table 4:** Comparison of background characteristics of patients with or without hypotension-related ADRs.

	Hypotension-Related ADRs (N = 37)	Non-Hypotension-Related ADRs (N = 108)	
All (N = 145)	25.5% (37/145)	74.5% (108/145)	Fisher’s test
Sex	Male/Female (73.0%/27.0%)	Male/Female (84.3%/15.7%)	
Male (N = 118)	27 (22.9%)	91 (77.1%)	*p* = 0.1454
Female (N = 27)	10 (37.0%)	17 (63.0%)	
History of NMIBC	Primary/Recurrent (67.6%/32.4%)	Primary/Recurrent (62.0%/38.0%)	
Primary (N = 92)	25 (27.2%)	67 (72.8%)	*p* = 0.6928
Recurrent (N = 53)	12 (22.6%)	41 (77.4%)	
ECOG PS	0/1 (100.0%/0.0%)	0/1 (97.2%/2.8%)	
0 (N = 142)	37 (26.1%)	105 (73.9%)	*p* = 0.5704
1 (N = 3)	0 (0.0%)	3 (100.0%)	
Complication of hypertension	Yes/No (37.8%/62.2%)	Yes/No (51.9%/48.1%)	
Yes (N = 70)	14 (20.0%)	56 (80.0%)	*p* = 0.1823
No (N = 75)	23 (30.7%)	52 (69.3%)	
Concomitant use of antihypertensive	Yes/No (35.1%/64.9%)	Yes/No (51.9%/48.1%)	
Yes (N = 62)	13 (21.0%)	49 (79.0%)	*p* = 0.3373
No (N = 83)	24 (28.9%)	59 (71.1%)	
Use from the day before administration to TURBT initiation	Yes/No (8.1%/91.9%)	Yes/No (3.7%/96.3%)	
Yes (N = 7)	3 (42.9%)	4 (57.1%)	*p* = 0.3717
No (N = 138)	34 (24.6%)	104 (75.4%)	
Type of anesthesia	General/Spinal (81.1%/18.9%)	General/Spinal (74.1%/25.9%)	
General anesthesia (N = 110)	30 (27.3%)	80 (72.7%)	*p* = 0.5058
Spinal anesthesia (N = 35)	7 (20.0%)	28 (80%)	
Age	≥80/<80 (18.9%/81.1%)	≥80/<80 (13.0%/87.0%)	
≥80 years (N = 21)	7 (33.3%)	14 (66.7%)	*p* = 0.5367
<80 years (N = 124)	30 (24.2%)	94 (75.8%)	
BMI	≥25/<25 (29.7%/70.3%)	≥25/<25 (34.3%/65.7%)	
≥25 (N = 48)	11 (22.9%)	37 (77.1%)	*p* = 0.6886
<25 (N = 97)	26 (26.8%)	71 (73.2%)	
eGFR (mL/min/1.73 m^2^)	<45/≥45 (10.8%/89.2%)	<45/≥45 (10.2%/89.8%)	
<45 (N = 15)	4 (26.7%)	11 (73.3%)	*p* = 1
≥45 (N = 130)	33 (25.4%)	97 (74.6%)	

Values are N (%) unless otherwise stated. Fisher’s test, Fisher’s exact test. ADR, adverse drug reaction; BMI, body mass index; NMIBC, non-muscle-invasive bladder cancer; ECOG PS, Eastern Cooperative Oncology Group Performance Status Scale; TURBT, transurethral resection of bladder tumor; eGFR, estimated glomerular filtration rate.

**Table 5:** Odds ratios and 95% confidence intervals of patient background factors for risk factors for hypotension-related ADRs.

	Odds Ratio (95% Confidence Interval)
Female sex (Ref: male)	1.983 (0.813–4.835)
Primary NMIBC (Ref: Recurrent NMIBC)	1.275 (0.578–2.810)
Without a complication of hypertension (Ref: with)	1.769 (0.824–3.799)
Without the use of antihypertensive (Ref: with)	1.533 (0.707–3.325)
With the use of antihypertensive from the day before administration to TURBT initiation (Ref: without)	2.294 (0.489–10.768)
General anesthesia (Ref: Spinal anesthesia)	1.496 (0.560–4.494)
Age ≥ 80 (Ref: <80)	1.567 (0.579–4.242)
BMI < 25 (Ref: ≥25)	1.232 (0.548–2.767)
eGFR ≥ 45 (Ref: <45)	1.069 (0.319–3.587)

ADR, adverse drug reaction; BMI, body mass index; NMIBC, non-muscle-invasive bladder cancer; TURBT, transurethral resection of bladder tumor; eGFR, estimated glomerular filtration rate.

**Table 6:** Comparison of the incidence and risk factors for hypotension between this study and the existing literature.

Definition	Author (Year)	Incidence of Hypotension	Risk Factor(s) for Hypotension
SBP ≤ 80 mmHg	This study	2.1% (3/145)	Not identified
SBP < 80 mmHg	Nohara et al. (2019) [14]	ND	Renin-angiotensin system inhibitor, General anesthesia
SBP < 80 mmHg	Shiratori et al. (2021) [7]	40.0% (10/26)	Hypertension, Antihypertensive
SBP ≤ 80 mmHg	Fukuhara et al. (2021) [8]	63.7% (156/245)	Hypertension, Calcium antagonist, General anesthesia
SBP < 80 mmHg	Nohara et al. (2024) [9]	75.6% (207/274)	Renal function impairment, General anesthesia (mild to moderate hypotension), Spinal anesthesia (severe hypotension)
SBP < 80 mmHg ^a^	Hori et al. (2025) [19]	61% (54/88) ^b^ 38% (10/26) ^c^	ND
SBP < 80 mmHg	Okada et al. (2026) [10]	69.5% (183/263)	Preoperative hemoglobin level (persistent hypotension)

ND, not described; SBP, systolic blood pressure; a, moderate to severe hypotension; b, administration < 4 h before TURBT; c, administration ≥ 4 h before TURBT.

## Data Availability

The datasets analyzed during this study are available from the corresponding author upon reasonable request.

## Supplementary Materials

The following supporting information can be downloaded at: https://www.mdpi.com/article/10.3390/life16050819/s1, Figure S1: Changes in the SBP for each patient. (A) All patients (N = 145); (B) Patients with hypotension-related ADRs (N = 37). (C) Three patients with decreased SBP ≤ 80 mmHg and mean SD SBP > 80 mmHg (N = 142); Figure S2: Correlation between systolic blood pressure (mmHg) and patient characteristics; Figure S3: Correlation between systolic blood pressure (% baseline) and patient background.

## Abbreviations

The following abbreviations are used in this manuscript:

5-ALA	5-aminolevulinic acid hydrochloride
AE	Adverse event
ADR	Adverse drug reaction
BMI	Body mass index
CI	Confidence interval
DBP	Diastolic blood pressure
PDD	Photodynamic diagnosis
PpIX	Protoporphyrin IX
PT	Preferred term
SBP	Systolic blood pressure
SD	Standard deviation
TURBT	Transurethral resection of bladder tumor
