# Supplementary material for: Blood Pressure Changes After Oral 5-Aminolevulinic Acid Hydrochloride Administered 4–8 h Before TURBT: An Additional Analysis of a Phase III Study (SPP2C102)
Source: Life (Basel). 2026 May 15;16(5):819. doi: 10.3390/life16050819 (PMC13209026; doi:10.3390/life16050819)
Supplement: Supplementary file 1 [file life-16-00819-s001.zip › life-4204736-supplementary.pdf]

Supplemental figures

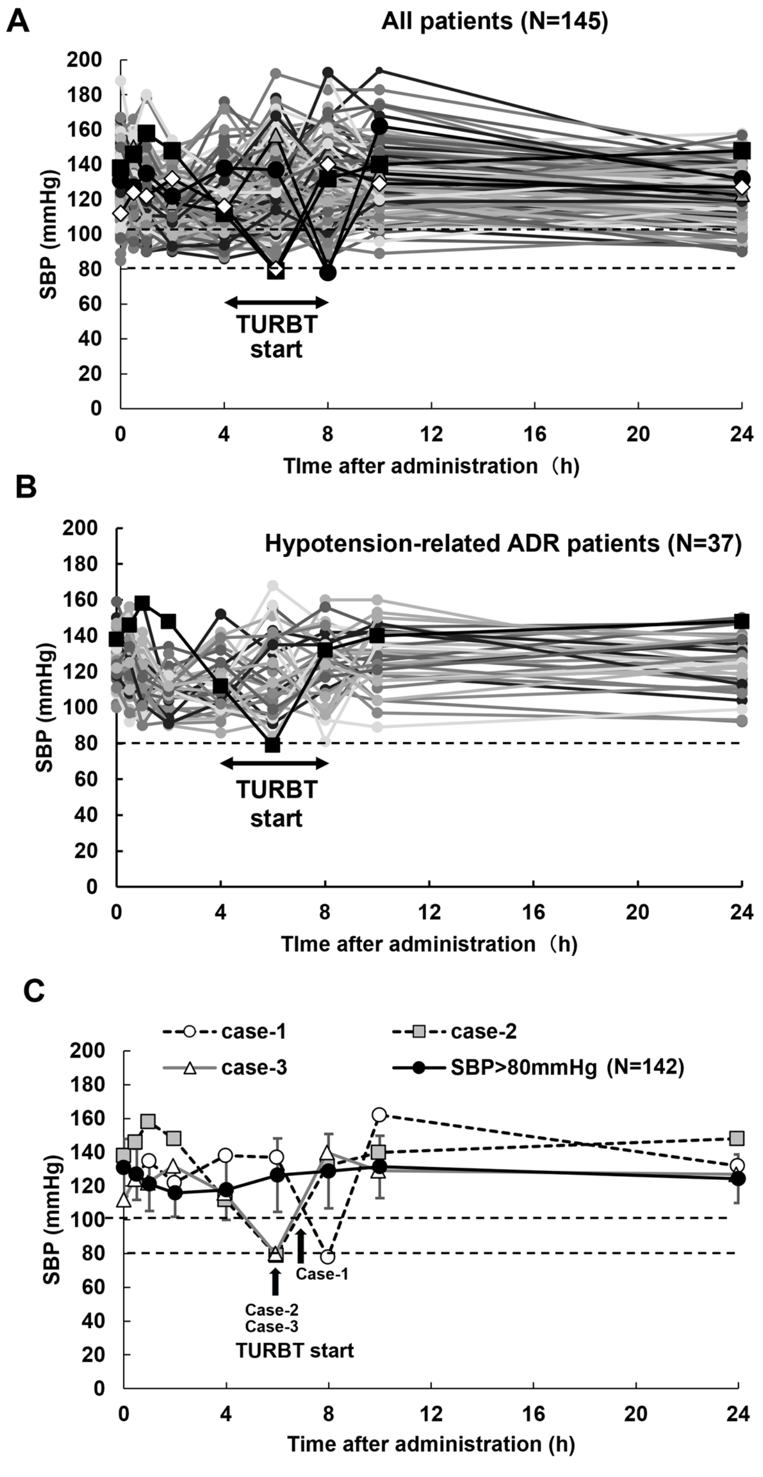

Figure S1. Changes in the SBP of each patient. (A) All patients (SAS) (N=145). (B) Patients with hypotension-ADR (N=37). (C) Three patients with SBP≤80 mmHg and mean SBP>80 mmHg (N=142). Horizontal dashed lines indicate 80 and 100 mmHg. The upward arrows indicate the time of TURBT initiation for each patient.

ADR, Adverse drug reaction; SBP, Systolic blood pressure; SAS, safety analysis set; TURBT, transurethral resection of bladder tumor; SD, standard deviation; SBP, systolic blood pressure. *, P<0.05; **, P<0.01 (t-test).

		SBP(mmHg)									
		0h	0.5h	1h	2h	4h	6h	8h	10h	24h	minimum
		correlation coefficient R									
Demographics	Sex	0.051	0.143	0.236	0.033	0.011	0.072	-0.003	0.200	0.062	0.026
	Age	0.195	0.103	0.038	0.053	0.053	0.167	0.133	0.181	0.020	0.081
	Height	-0.169	-0.083	0.052	-0.003	-0.087	-0.096	-0.130	0.035	0.071	-0.110
	Weight	0.043	0.098	0.185	0.074	0.068	0.000	0.060	0.149	0.290	0.039
	BMI	0.180	0.192	0.197	0.095	0.150	0.094	0.165	0.173	0.327	0.135
	Body temperature	-0.043	-0.052	-0.059	0.040	0.066	-0.068	0.040	-0.102	-0.057	0.056
	pulse rate	0.101	0.051	0.078	0.032	0.108	-0.140	0.064	0.084	0.122	-0.001
procedure	TURBT start time	-0.023	0.010	0.012	0.005	0.111	0.131	-0.071	0.057	0.103	-0.012
	Infusion dosage before TURBT (ml)	-0.035	-0.118	-0.124	-0.136	-0.059	-0.085	-0.043	-0.062	0.049	-0.039
	5-ALA exposure before TURBT (h)	-0.144	-0.102	-0.011	-0.014	-0.153	-0.182	-0.163	-0.018	0.078	-0.164
	Study drug administration time	0.029	0.047	0.017	0.011	0.161	0.191	-0.011	0.064	0.072	0.047
	Drinking time before TURBT	0.006	-0.043	-0.088	-0.042	0.016	0.087	-0.088	0.093	0.048	-0.083
	water deprivation time	0.087	0.104	0.027	-0.001	0.009	0.112	0.054	0.207	0.221	0.071
	Type of anesthesia	-0.079	-0.125	-0.097	-0.017	0.032	0.093	0.117	0.072	-0.068	-0.002
Name of anesthetic	Propofol	-0.019	-0.005	-0.018	-0.010	0.118	0.052	0.083	0.011	-0.106	-0.028
	Sevoflurane	-0.075	-0.080	-0.147	-0.055	-0.039	0.062	0.004	0.002	-0.141	-0.045
	Desflurane	-0.056	0.023	0.083	-0.029	0.096	-0.039	0.055	-0.024	0.065	-0.010
	Anerem	-0.025	-0.116	-0.061	0.008	-0.117	0.082	0.061	0.107	0.115	0.062
	markaine	0.065	0.118	0.083	0.005	-0.031	-0.086	-0.112	-0.079	0.046	-0.014
	Anapain	-0.016	-0.028	-0.016	-0.094	0.075	-0.024	0.064	0.080	-0.038	0.011
	Doloreptan	0.021	0.004	-0.038	-0.067	-0.023	-0.017	-0.003	-0.041	-0.012	0.016
	Buvivacaine	0.075	0.044	0.074	0.064	-0.004	-0.041	-0.032	0.029	0.119	0.079
	Antihypertensive	-0.034	0.016	-0.021	0.016	0.035	-0.108	0.116	0.069	0.103	0.071
complications	Diabetes	-0.040	-0.007	-0.047	-0.092	-0.039	-0.073	-0.028	0.002	0.080	-0.050
	Hyperlipidemia	0.140	0.025	0.029	0.069	0.135	0.162	0.094	0.136	0.208	0.018
	dyslipidemia	-0.038	-0.091	-0.060	-0.063	-0.084	0.029	-0.083	-0.084	-0.018	-0.002
	Hyperuricemia	0.076	-0.004	-0.046	-0.079	0.069	0.072	0.158	0.124	0.107	0.013
	Hypertension	0.350	0.354	0.210	0.192	0.242	0.193	0.298	0.365	0.289	0.298
	hematuria	0.074	0.140	0.122	-0.093	-0.024	-0.008	0.026	0.047	0.016	-0.044
	osteoporosis	0.067	0.142	0.087	0.049	-0.022	0.014	-0.024	-0.161	-0.042	-0.034
	cataract	0.068	0.075	0.080	0.070	0.024	-0.007	0.026	0.035	0.017	-0.054
	Prostatic hypertrophy	0.097	0.008	-0.043	0.017	0.026	0.066	-0.021	0.176	0.142	0.016
	Chronic gastritis	0.049	0.064	0.030	-0.051	-0.026	-0.107	0.104	0.061	0.055	0.018
	sleep apnea syndrome	-0.021	0.075	0.245	0.073	0.050	-0.191	0.024	0.008	0.110	-0.106
	spinal canal stenosis	-0.020	-0.056	-0.008	0.020	-0.145	0.097	-0.162	-0.011	-0.147	-0.137
	constipation	0.001	-0.068	-0.027	-0.029	0.012	-0.072	0.101	-0.022	-0.143	-0.018
	insomnia	0.084	-0.006	-0.009	0.105	0.114	-0.023	-0.006	0.075	0.152	0.101
	anemia	-0.055	-0.021	0.023	0.086	0.062	0.096	0.202	0.152	-0.124	0.065
	vomiting, nausea	0.111	0.033	0.004	0.081	0.047	0.095	-0.049	-0.163	0.042	0.070
Hematology	white blood cell count	0.078	0.103	0.119	-0.023	-0.086	-0.037	-0.094	0.041	0.168	-0.013
	Number of red blood cells	0.039	0.070	0.140	0.056	0.073	-0.045	-0.153	-0.010	0.146	-0.015
	hemoglobin amount	0.049	0.067	0.166	0.056	0.058	-0.027	-0.178	0.010	0.108	-0.009
	hematocrit value	0.026	0.066	0.163	0.051	0.062	-0.026	-0.170	-0.019	0.116	-0.013
	platelet count	-0.017	0.015	0.006	-0.047	-0.003	-0.022	-0.024	-0.092	-0.023	0.045
	Neutrophil count	0.112	0.196	0.228	0.084	0.179	0.052	-0.018	-0.001	0.050	0.089
	lymphocyte count	-0.119	-0.213	-0.239	-0.088	-0.186	-0.065	-0.017	-0.018	-0.067	-0.086
	monocyte count	0.052	0.015	0.004	0.049	-0.003	0.060	0.100	0.083	0.088	0.040
	Eosinophil count	-0.125	-0.101	-0.126	-0.124	-0.128	-0.079	0.006	-0.037	-0.094	-0.128
	Basophil count	-0.065	-0.097	-0.171	0.024	0.039	0.037	0.074	-0.008	-0.019	-0.044
Blood biochemistry	AST	0.078	-0.043	0.006	-0.044	0.197	0.067	0.027	0.020	-0.023	0.027
	ALT	0.122	0.021	0.082	-0.022	0.188	0.069	0.072	0.125	0.081	-0.053
	ALP	0.055	0.057	0.052	-0.128	-0.043	-0.031	-0.016	-0.019	0.026	-0.105
	γ-GTP	0.132	0.062	0.091	0.143	0.228	0.013	0.157	0.070	0.156	0.110
	total protein	0.020	-0.064	-0.012	-0.103	0.025	0.070	-0.095	-0.112	0.014	0.012
	albumin	-0.040	-0.092	-0.033	-0.094	-0.025	-0.067	-0.056	-0.151	-0.061	-0.010
	amylase	-0.018	0.013	0.137	0.037	-0.103	-0.049	0.046	0.009	-0.086	0.038
	bilirubin	0.145	0.140	0.128	0.172	0.172	0.121	-0.073	0.103	-0.003	0.145
	Blood glucose level	0.099	0.075	0.006	-0.053	0.137	0.029	0.133	0.058	0.054	0.063
	total cholesterol	-0.029	-0.080	-0.048	0.049	0.147	0.040	-0.054	-0.072	-0.025	0.015
	HDL cholesterol	0.054	0.072	0.009	0.138	0.139	-0.012	0.016	-0.097	-0.102	0.067
	triglycerides	0.013	-0.066	0.013	-0.164	0.032	0.017	-0.010	0.086	0.169	-0.017
	CRP	-0.194	-0.172	-0.094	-0.133	-0.154	-0.150	-0.200	-0.036	0.024	-0.167
	LDH	-0.027	-0.010	-0.025	-0.058	-0.005	-0.030	-0.019	-0.116	-0.048	0.025
	BUN	0.035	0.081	0.055	0.067	0.072	0.113	0.165	0.131	-0.013	0.118
	creatinine	0.097	0.144	0.146	0.051	0.036	0.105	0.095	0.201	0.108	0.103
	uric acid	0.049	0.027	0.101	0.115	0.224	0.058	0.129	0.192	0.080	0.111
	sodium	0.049	0.085	-0.050	0.108	0.036	0.071	0.063	0.136	0.095	-0.009
	potassium	-0.124	-0.067	-0.001	-0.098	-0.156	-0.064	-0.095	-0.101	-0.063	-0.037
	chloride	0.091	0.147	0.005	0.115	0.029	0.001	0.069	0.160	0.120	-0.012
	eGFR	-0.160	-0.127	-0.076	-0.075	-0.035	-0.120	-0.096	-0.195	-0.113	-0.066
Urinalysis	urine protein	0.026	0.144	0.043	-0.024	0.000	-0.043	0.112	0.066	0.039	0.023
	urine sugar	0.072	-0.002	-0.044	-0.054	0.044	-0.052	-0.051	-0.044	-0.049	0.001
	Urobilinogen	0.026	0.077	0.017	-0.019	-0.075	-0.026	-0.010	-0.116	0.020	-0.014
	urinary occult blood	0.177	0.128	-0.050	-0.101	-0.012	0.069	0.108	0.083	0.014	0.003

0.2 ≤ R < 0.4   -0.4< R ≤ -0.2

Figure S2. Correlation between systolic blood pressure (mmHg) and various factors.

BMI, body mass index; TURBT, transurethral resection of bladder tumor; AST, aspartate amino transferase; ALT, alanine amino transferase; γ-GTP, γ-glutamyl-transpeptidase; HDL, high-density lipoprotein; CRP, C reactive protein; LDH, lactate dehydrogenase; BUN, blood urea nitrogen; eGFR, estimated glomerular filtration rate.

		SBP(% Baseline)								
		0.5h	1h	2h	4h	6h	8h	10h	24h	minimum
		correlation coefficient R								
Demographics	Sex	0.089	0.192	-0.043	-0.049	0.021	-0.054	0.146	-0.026	-0.062
	Age	-0.122	-0.174	-0.148	-0.111	0.045	-0.002	0.002	-0.182	-0.130
	Height	0.095	0.224	0.146	0.046	0.007	-0.024	0.179	0.245	0.106
	Weight	0.030	0.133	-0.020	0.008	-0.057	0.008	0.094	0.186	-0.031
	BMI	-0.029	0.002	-0.139	-0.027	-0.058	0.020	-0.005	0.060	-0.118
	Body temperature	-0.007	-0.027	0.073	0.108	-0.038	0.065	-0.071	0.013	0.094
	pulse rate	-0.065	-0.021	-0.074	0.025	-0.215	-0.020	-0.007	-0.070	-0.164
procedure	TURBT start time	0.016	0.007	0.002	0.126	0.148	-0.072	0.053	0.061	-0.047
	Infusion dosage before TURBT (ml)	-0.097	-0.096	-0.079	-0.028	-0.063	-0.019	-0.023	0.072	0.004
	5-ALA exposure before TURBT (h)	0.042	0.129	0.126	-0.054	-0.099	-0.065	0.109	0.130	-0.062
	Study drug administration time	0.001	-0.038	-0.042	0.141	0.179	-0.046	0.014	0.014	-0.023
	Drinking time before TURBT	-0.055	-0.101	-0.054	0.014	0.096	-0.080	0.087	0.050	-0.050
	water deprivation time	0.011	-0.062	-0.087	-0.076	0.046	-0.011	0.125	0.072	-0.040
	Type of anesthesia	-0.058	-0.020	0.055	0.093	0.152	0.154	0.142	-0.008	0.034
Name of anesthetic	Propofol	0.015	0.003	0.008	0.136	0.060	0.087	0.040	-0.088	-0.036
	Sevoflurane	0.006	-0.064	0.044	0.029	0.124	0.066	0.091	-0.002	0.090
	Desflurane	0.087	0.139	0.009	0.149	-0.009	0.077	0.006	0.015	-0.065
	Anerem	-0.119	-0.044	0.026	-0.112	0.112	0.066	0.110	0.115	0.071
	markaine	0.067	0.021	-0.051	-0.079	-0.135	-0.140	-0.135	0.004	-0.033
	Anapain	-0.019	-0.006	-0.084	0.093	-0.019	0.067	0.088	-0.021	0.019
	Doloreptan	-0.019	-0.054	-0.082	-0.051	-0.027	-0.021	-0.053	-0.020	0.005
	Buvivacaine	-0.045	-0.004	-0.020	-0.074	-0.094	-0.080	-0.041	0.023	-0.007
	Antihypertensive	0.052	-0.001	0.033	0.065	-0.093	0.132	0.094	0.110	0.079
complications	Diabetes	0.039	-0.010	-0.062	-0.005	-0.063	-0.009	0.032	0.115	0.000
	Hyperlipidemia	-0.150	-0.128	-0.082	0.004	0.054	-0.024	-0.009	0.040	-0.107
	dyslipidemia	-0.077	-0.038	-0.033	-0.052	0.061	-0.052	-0.059	0.038	0.047
	Hyperuricemia	-0.117	-0.142	-0.166	-0.010	0.000	0.088	0.045	0.024	-0.049
	Hypertension	-0.025	-0.147	-0.187	-0.066	-0.064	0.040	0.036	-0.128	-0.129
	hematuria	0.053	0.038	-0.173	-0.103	-0.068	-0.041	-0.031	-0.280	-0.334
	osteoporosis	0.067	0.013	-0.032	-0.084	-0.045	-0.072	-0.227	-0.087	-0.079
	cataract	0.003	0.011	-0.005	-0.021	-0.055	-0.025	-0.021	-0.030	-0.089
	Prostatic hypertrophy	-0.121	-0.149	-0.079	-0.066	-0.008	-0.097	0.085	0.041	-0.056
	Chronic gastritis	0.014	-0.024	-0.104	-0.079	-0.146	0.068	0.009	0.003	-0.028
	sleep apnea syndrome	0.105	0.284	0.078	0.069	-0.190	0.028	0.017	0.105	-0.064
	spinal canal stenosis	-0.058	-0.004	0.032	-0.145	0.116	-0.148	0.016	-0.090	-0.080
	constipation	-0.067	-0.025	-0.034	0.016	-0.074	0.101	-0.030	-0.095	-0.001
	insomnia	-0.133	-0.115	-0.010	0.024	-0.097	-0.075	-0.018	0.036	-0.006
	anemia	0.057	0.102	0.165	0.118	0.143	0.232	0.209	-0.036	0.118
	vomiting, nausea	-0.112	-0.118	-0.038	-0.055	-0.001	-0.124	-0.266	-0.041	-0.025
Hematology	white blood cell count	0.034	0.057	-0.100	-0.170	-0.096	-0.146	-0.023	-0.001	-0.148
	Number of red blood cells	0.021	0.098	-0.017	0.028	-0.092	-0.187	-0.060	0.095	-0.040
	hemoglobin amount	0.009	0.124	-0.014	0.003	-0.083	-0.214	-0.046	0.042	-0.054
	hematocrit value	0.037	0.144	0.003	0.028	-0.066	-0.194	-0.055	0.073	-0.034
	platelet count	0.046	0.031	-0.027	0.009	0.002	-0.024	-0.078	-0.133	-0.088
	Neutrophil count	0.092	0.127	-0.050	0.088	-0.027	-0.102	-0.112	-0.136	-0.120
	lymphocyte count	-0.097	-0.130	0.054	-0.085	0.020	0.076	0.100	0.144	0.141
	monocyte count	-0.054	-0.055	-0.012	-0.061	0.017	0.060	0.038	0.045	0.012
	Eosinophil count	0.028	0.002	0.030	-0.022	0.010	0.089	0.084	0.024	0.007
	Basophil count	-0.060	-0.124	0.088	0.092	0.078	0.098	0.041	0.011	-0.009
Blood biochemistry	AST	-0.156	-0.080	-0.124	0.142	0.015	-0.035	-0.060	-0.066	-0.034
	ALT	-0.145	-0.051	-0.154	0.080	-0.030	-0.034	0.008	-0.009	-0.118
	ALP	0.001	0.000	-0.180	-0.107	-0.058	-0.069	-0.062	-0.005	-0.115
	γ-GTP	-0.112	-0.051	-0.017	0.109	-0.095	0.039	-0.061	0.015	-0.031
	total protein	-0.119	-0.055	-0.152	0.005	0.059	-0.113	-0.141	0.029	0.033
	albumin	-0.080	-0.017	-0.075	0.014	-0.038	-0.027	-0.119	0.043	0.092
	amylase	0.052	0.174	0.075	-0.088	-0.038	0.071	0.043	-0.097	0.016
	bilirubin	-0.040	-0.033	0.004	0.048	0.022	-0.185	-0.049	-0.129	-0.023
	Blood glucose level	-0.043	-0.103	-0.169	0.049	-0.063	0.048	-0.048	-0.037	-0.036
	total cholesterol	-0.075	-0.025	0.074	0.192	0.056	-0.026	-0.053	0.041	0.080
	HDL cholesterol	0.013	-0.049	0.098	0.112	-0.047	-0.011	-0.143	-0.108	0.033
	triglycerides	-0.105	0.002	-0.187	0.016	-0.004	-0.028	0.064	0.124	-0.021
	CRP	0.061	0.108	0.053	-0.017	-0.013	-0.043	0.158	0.198	0.058
	LDH	0.016	-0.003	-0.044	0.015	-0.014	-0.008	-0.108	-0.012	0.041
	BUN	0.066	0.026	0.036	0.067	0.094	0.146	0.110	0.011	0.121
	creatinine	0.073	0.079	-0.030	-0.043	0.043	0.039	0.134	0.016	0.027
	uric acid	-0.053	0.041	0.038	0.186	0.008	0.079	0.144	-0.027	-0.007
	sodium	0.024	-0.131	0.041	-0.012	0.026	0.022	0.092	0.113	0.031
	potassium	0.081	0.141	0.032	-0.047	0.024	-0.007	0.012	0.068	0.091
	chloride	0.070	-0.080	0.027	-0.052	-0.065	0.015	0.093	0.088	0.006
	eGFR	0.036	0.071	0.071	0.105	-0.016	0.004	-0.067	0.035	0.056
Urinalysis	urine protein	0.162	0.039	-0.042	-0.020	-0.060	0.101	0.059	0.063	0.030
	urine sugar	-0.086	-0.115	-0.121	-0.013	-0.116	-0.099	-0.109	-0.091	-0.052
	Urobilinogen	0.051	-0.013	-0.049	-0.109	-0.049	-0.031	-0.141	-0.008	-0.034
	urinary occult blood	-0.082	-0.249	-0.292	-0.195	-0.079	-0.024	-0.088	-0.119	-0.135

(pink) $0.2 \leq R < 0.4$ (blue) $-0.4 < R \leq -0.2$

Figure S3. Correlation between systolic blood pressure (% baseline) and various factors.

BMI, body mass index; TURBT, transurethral resection of bladder tumor; AST, aspartate amino transferase; ALT, alanine amino transferase; γ-GTP, γ-glutamyl-transpeptidase; HDL, high-density

lipoprotein; CRP, C reactive protein; LDH, lactate dehydrogenase; BUN, blood urea nitrogen; eGFR, estimated glomerular filtration rate.
